# Biomarkers for systemic lupus erythematosus: A scoping review

**DOI:** 10.1002/iid3.70022

**Published:** 2024-10-04

**Authors:** Su‐jie Zhang, Rui‐yang Xu, Long‐li Kang

**Affiliations:** ^1^ Key Laboratory for Molecular Genetic Mechanisms and Intervention Research on High Altitude Disease of Tibet Autonomous Region, Key Laboratory of High Altitude Environment and Genes Related to Diseases of Tibet Autonomous Region School of Medicine, Xizang Minzu University Xianyang Shaanxi China

**Keywords:** biomarkers, diagnosis, disease activity, lupus nephritis, monitoring, neuropsychiatric in SLE, systemic lupus erythematosus

## Abstract

**Background:**

In recent years, newly discovered potential biomarkers have great research potential in the diagnosis, disease activity prediction, and treatment of systemic lupus erythematosus (SLE).

**Objective:**

In this study, a scoping review of potential biomarkers for SLE over several years has identified the extent to which studies on biomarkers for SLE have been conducted, the specificity, sensitivity, and diagnostic value of potential biomarkers of SLE, the research potential of these biomarkers in disease diagnosis, and activity detection is discussed.

**Methods:**

In PubMed and Google Scholar databases, “SLE,” “biomarkers,” “predictor,” “autoimmune diseases,” “lupus nephritis,” “neuropsychiatric SLE,” “diagnosis,” “monitoring,” and “disease activity” were used as keywords to systematically search for SLE molecular biomarkers published from 2020 to 2024. Analyze and summarize the literature that can guide the article.

**Conclusions:**

Recent findings suggest that some potential biomarkers may have clinical application prospects. However, to date, many of these biomarkers have not been subjected to repeated clinical validation. And no single biomarker has sufficient sensitivity and specificity for SLE. It is not scientific to choose only one or several biomarkers to judge the complex disease of SLE. It may be a good direction to carry out a meta‐analysis of various biomarkers to find SLE biomarkers suitable for clinical use, or to evaluate SLE by combining multiple biomarkers through mathematical models. At the same time, advanced computational methods are needed to analyze large data sets and discover new biomarkers, and strive to find biomarkers that are sensitive and specific enough to SLE and can be used in clinical practice, rather than only staying in experimental research and data analysis.

## 1 INTRODUCTION

Systemic lupus erythematosus (SLE) is a chronic autoimmune disease characterized by autoantibody production, inflammation, and tissue damage in multiple organs resulting from the activation of numerous preinflammatory pathways.^1^, ^2^ The incidence of SLE is 0.3–31.5 in 100,000 per year, and the adjusted prevalence is approaching, or even exceeding, 50–100 in 100,000.^3^ The overall age‐adjusted/sex‐adjusted incidence was 7.4 per 100,000 persons/year, with stabilizing trends in women but increasing in men, and an average (±SD) age of diagnosis at 43 (±15) years.^3^ In adults, SLE is more common in women, but 15%–20% of SLE may occur in childhood. Moreover, the clinical manifestations of SLE in children are more severe than those in adults.^4^ The incidence of SLE is increasing year by year, but the mortality rate has decreased. A 2006–2016 study on the mortality of hospitalized patients with SLE in the USA showed that the risk of in‐hospital death of SLE patients decreased from 2.2% to 1.5%, and the mortality rate decreased significantly. At the same time, by comparison, Black, Hispanics, and Asian/Pacific Islanders with SLE had higher mortality.^5^

SLE is a chronic autoimmune disease that mainly affects women but is usually more severe and has a worse prognosis in male patients.^6^ The main causes of death in patients with SLE are end‐stage disease, severe infection, and cardiovascular complications, and the frequency of occurrence is different in different countries and regions.^7^, ^8^, ^9^ There are some differences in the causes of death of SLE in China. The proportion of infection and lupus encephalopathy is higher, while the proportion of cardiovascular complications is lower. Ethnicity and more aggressive treatment may be responsible for the difference in the composition of deaths.^10^

In recent years, biomarkers have been widely employed in the diagnosis, prediction, assessment, and management of SLE, diabetes, heart disease, cancer, and other diseases.^11^ Biomarkers are used to monitor disease development before the diagnosis of the disease, during diagnosis, they can assist in determining staging, grading, and primary treatment options, and after the diagnosis, to monitor treatment, complementary treatment options, or to monitor recurrent disease.^12^, ^13^, ^14^ Some researchers have also proposed the definition of an ideal biomarker for SLE—a tool to help doctors make diagnosis and treatment decisions.^15^ This biomarker should be specific, easy to detect, track disease activity, useful in follow‐up, and reasonably priced. However, due to the complexity of the disease and the diversity of the etiologies, it is sometimes difficult to distinguish SLE from other similar diseases. Therefore, screening and identifying reliable markers and revealing potential mechanisms of action are crucial for better diagnosis and effective treatment of SLE.

## 2 METHODS

In PubMed and Google Scholar databases, “SLE,” “biomarkers,” “predictor,” “autoimmune diseases,” “lupus nephritis,” “neuropsychiatric SLE,” “diagnosis,” “monitoring,” and “disease activity” were used as keywords to systematically search for SLE molecular biomarkers published from 2020 to 2024. Preliminary screening. According to whether the experimental data of the markers have available value and participate in the pathogenesis of SLE, the retrieved literature is analyzed and information is extracted to see whether it can be used for article writing. Tables were made according to diagnosis, disease activity, and others based on “organ‐specific,” “marker,” “specimen,” “method,” and “key point.” At the same time, the available experimental data and participation mechanisms of potential biomarkers of SLE involving organs are classified and summarized.

## 3 RESULTS

Further analysis and data collation of potential candidate biomarkers are shown in Tables 1, 2, 3.

**TABLE 1:** Potential diagnostic markers for SLE.

Organ‐ specific	Marker	Specimen	Method	Key points	Reference
	Circulating exosomal microRNAs	Serum	PCR*	AUC* of 0.790 for exosomal miR21, AUC of 0.709 for exosomal miR‐155.	[16]
	DDX60*	Blood	RT‐qPCR*	The AUC for predicting high disease activity of SLE was 0.8818.	[17]
	Serum leptin	Serum	ELISA*	AUC = 0.972, when the cut‐off value was 9.9 ng/mL, the accuracy, specificity, sensitivity, NPV*, and PPV* of leptin were 90.8%, 92%, 90%, 86.8%, and 94%, respectively.	[18]
	Adiponectin	Serum	ELISA	AUC = 0.833, when the cut‐off value was 9.4 ng/mL, the accuracy of adiponectin was 80.8%, the specificity was 82%, the sensitivity was 80%, the NPV was 74.5%, and the PPV was 86.2%.	[18]
	Anaerococcus, Gardnerella, Lactobacillus	Feces	PCR	The probability of difference between SLE and HC* among Anaerococcus, Gardnerella and Lactobacillus was 82.80–83.60%.	[19]
	Bacteroides, Escherichia‐Shigella, Streptococcus	Vagina	PCR	The accuracy of the identification of Bacteroides, Escherichia‐Shigella, and Streptococcus between SLE patients and HC patients was 89.90% − 100.00%.	[19]
	HMGB1(NRGs*)	WB*, PBMC*	The correlation and protein–protein interaction analyses	AUCs of 0.930 for HMGB1, showed its potency as useful diagnostic biomarkers	[20]
	ITGB2(NRGs)	WB, PBMC	The correlation and protein–protein interaction analyses	AUCs of 0.901 for ITGB2, showed its potency as useful diagnostic biomarkers	[20]
	CREB5(NRGs)	WB, PBMC	The correlation and protein–protein interaction analyses	AUCs of 0.788 for CREB5, CREB5 is involved in PI3K‐Akt and Toll‐like receptor signaling pathways leading to SLE.	[20]
	IgA*	Saliva	ELISA	The salivary IgA subtype is associated with disease, with an AUC of 0.855 for IgA1 and 0.761 for IgA2.	[21]
	miR‐342‐3p	Serum	qRT‐PCR*	The expression of miR‐342‐3p in SLE patients was significantly lower than that in healthy people.	[22]
	Sema4A*	Serum	ELISA	Sema4A is positively correlated with SLEDAI and has high diagnostic value for SLE.	[23]
	sTREM‐1*	Serum	PCR, ELISA	Serum sTREM‐1 was significantly elevated in SLE patients, with AUC = 0.9511.	[24]
	IFI44L*	PBMC	RT‐qPCR	ROC* analysis showed that IFI44L had diagnostic significance for SLE.	[25]
	RGC‐32*	Serum	ELISA	AUC = 0.803, when RGC‐32 ≥ 206.4 pg/mL, the specificity and sensitivity of SLE diagnosis were 85% and 77.5%.	[26]
	His*	Plasma	RNA‐seq*	Data analysis showed that His could effectively identify SLE.	[27]
	cf‐eccDNA*	Blood	DifCir*	The number of eccDNA in the healthy control group was lower than that in DNASE1L3* deficient SLE patients, and the eccDNA was reduced by 0.0321 times.	[28]
	IFIT3*\MX1*\ TOMM40*\STAT1* STAT2*\OAS3*	PBMC	ELISA	AUC = 0.723 (95% CI = 0.591–0.854)	[29]
	S100A8*	Serum	ELISA	AUC = 0.831 (95% CI, 0.765–0.897) Sensitivity: 61% Specificity: 91.1% PPV: 95.7%	[30]
	S100A8	Urine	ELISA	AUC = 0.751 (95% CI, 0.648–0.854) Sensitivity: 99% Specificity: 55.6% PPV: 63.9%	[30]
	S100A8	Salivary	ELISA	AUC = 0.729 (95% CI, 0.646–0.812). Sensitivity: 52% Specificity: 91.1% PPV: 87.3%	[30]
	ABCB1	PBMC	qRT‐PCR*	AUC = 0.754	[31]
	IFI27	PBMC	qRT‐PCR	The diagnostic effect and discovered that the AUC values of the biomarkers, IFI27 were 0.875, *p* = .2746	[31]
	PLSCR1	PBMC	qRT‐PCR	The diagnostic effect and discovered that the AUC values of the biomarkers, PLSCR1 were 0.851, *p* = .1376	[31]
	lncRNA SNHG1	PBMCs	RT‐qPCR	SNHG1 expression was positively correlated with SLEDAI score, IgG, CRP, and ESR, and negatively correlated with C3 and C4.	[32]
	KLRB1, KLRF1, GZMK, IL‐7R and CD40LG	PBMC	DEGs analysis	SVM* and LASSO regression analysis showed that KLRB1, KLRF1, GZMK, IL‐7R, and CD40LG had good diagnostic ability.	[33]
	MINA* 53 protein	Blood	ELISA, real‐time PCR	When Mina53 serum level was 125.4, AUC = 0.951 and 8.5, AUC = 0.88, the sensitivity and specificity of SLE diagnosis were the highest.	[34]
LN	Urinary exosome tsRNAs*	Urine	RT‐PCR	tRF3‐Ile‐AAT‐1: AUC = 0.777 (specificity 66.69%, sensitivity 79.63%) TirNA5‐LYS‐CT‐1: AUC = 0.715 (specificity 76.92%, sensitivity 66.96%)	[35]
LN	Urine sTREM‐1*	Blood, urine	ELISA	Urinary sTREM‐1 level in SLE was higher than that in healthy group, and was positively correlated with renal sledai score, negatively correlated with serum C3 and C4 levels, and positively correlated with albuminuria.	[36]
LN	VSIG4*	Serum	Quantitative protein microarray	AUC of 0.93 for VSIG4.	[37]
LN	EGFR, FOLR2, PDGF‐RB, and TFRC	Renal biopsy	scRNA sequence data analysis and immunohistochemistry	EGFR, FOLR2, PDGF‐RB, and TFRC have the potential to be novel LN biomarkers, but the expression profiles have not been confirmed.	[38]
LN	BCDF*	Serum	ELISA	Increased levels of BCDF in SLE patients Sensitivity 80.6%, Specificity 70.8%	[39]
LN	IgM*	Serum	ELISA	Increased levels of IgM in SLE patients Sensitivity 97.2%, Specificity 87.5%	[39]
LN	GDF‐15*	Serum	ELISA	GDF‐15 was related to SLE pathogenesis, Sensitivity 0.907, Specificity 0.800	[40]
LN	MX2*	Whole blood and peripheral blood	qRT‐PCR	The ROC curve for diagnostic efficacy validation of MX2 with AUCs of 0.958 (GSE121239) and 0.9769 (GSE11907)	[41]
LN	IFI44*	PBMC	qRT‐PCR	The AUC of IFI44 was 0.850, the diagnostic specificity was 0.850, and the sensitivity was 0.923.	[42]
LN	Adiponectin	Urine	ELISA	(18,000 pg/mL) Sensitivity: 91.7% (95% CI) Specificity: 90.9% (95% CI), PPV: 52.4% (95% CI)	[43]
LN	MCP‐1	Urine	ELISA	(1341 pg/mL) Sensitivity: 37.5% (95% CI) Specificity: 97.3% (95% CI) PPV: 60.0% (95% CI)	[43]
LN	sVCAM‐1	Urine	ELISA	(46,000 pg/mL) and (10,3700 pg/mL) Sensitivity: 79.2% and 66.7% (95% CI) Specificity 81.1% and 95.5% (95% CI) PPV 31.2% and 31.2% (95% CI)	[43]
LN	PF4	Urine	ELISA	(134 pg/mL) Sensitivity: 83.3% (95% CI) Specificity: 93.7% (95% CI) PPV: 58.8% (95% CI)	[43]
LN	PHACTR4*	Serum	ELISA	AUC: 0.99, PHACTR4 ICx* were significantly elevated	[44]
LN	P3H1*	Serum	ELISA	AUC: 0.82, P3H1 ICx was found significantly downregulated in LN	[44]
LN	RGS12*	Serum	ELISA	AUC: 0.90, RGS12 ICx was found upregulated in LN	[44]
j‐NPSLE	neopterin	CSF*	Liquid chromatography	Neopterin levels were significantly elevated in both active and inactive NPSLE patients.	[45]
j‐NPSLE	IFN‐α*	CSF	ELISA	It was significantly increased in both active and inactive NPSLE patients.	[45]
JSLE*	hsa_circ_0008945	PBMCs	RT‐qPCR	AUC = 0.790 (95%CI: 0.6733–0.9067, *p* < .001), specificity 83.33%, sensitivity 70%	[46]
pSLE*	Ang*−1, Ang‐2, and Tie2	Serum urine	ELISA	The AUC values of Ang‐1, Ang‐2, and Tie2 in serum and urine were all greater than 0.7.	[47]
cSLE	IFI44L* promoter methylation	Blood	HRM‐qPCR	AUC = 0.867, specificity 1.000, sensitivity 0.753.	[48]
NPSLE*	sNfL*	Serum	SiMoA*	AUC = 0.646 (95% CI: 0.554–0.738, *p* = .003)	[49]
NPSLE	α‐Klotho*	CSF	ELISA	AUC = 0.94 (*p* < .001)	[50]
NPSLE	l‐Selectin*	CSF	1000‐plexed proteins array	Sensitivity: 62.16%; specificity: 72.22%	[51]
NPSLE	Trappin‐2	CSF	1000‐plexed proteins array	Sensitivity: 89.19%; specificity: 66.67%	[51]
NPSLE	TCN2	CSF	1000‐plexed proteins array	AUC = 0.703, *p* = .0155 and sensitivity: 54.05%; specificity: 88.89%	[51]
NPSLE	CST6	CSF	1000‐plexed proteins array	AUC = 0.738, *p* = .0043 and sensitivity: 51.35%; specificity: 94.44%	[51]
NPSLE	MAP‐2*	CSF	ELISA	The positive rate of anti‐MAP‐2 antibody in NPSLE was 33.3% (8/24), the positive rate of CSF showed high specificity for NPSLE.	[52]
SLE‐PAH*	GDF‐15*	Serum	Enzyme‐linked immunosorbent assay	AUC = 0.84, when GDF‐15 ≥ 733 pg/mL, the specificity, and sensitivity of SLE‐PAH diagnosis were 58.6% and 91%.	[53]
IMN*	PLA2R‐AB*	Serum	ELISA	ROC curves of the PLA2R‐AB showed that AUC: 0.907, Sensitivity 94.3%, Specificity 82.1%.	[54]
CVD*	Lactosylceramides	Plasma	HPLC‐MS/MS*	Lactosylceramides correlate negatively with plaque area and C3	[55]

* Ang, angiopoietins; AUC, area under the ROC curve; BCDF, B cell differentiating factor; cf‐eccDNA, cell‐free (cf) extrachromosomal circular DNA (eccDNA); CI, chronicity index; CSF, cerebrospinal fluid; C3, complement C3; C4, complement C4; CSF proteins: TCN2, CST6, lL‐selectin,‐selectin, Trappin‐2; CVD, cardiovascular disease; DDX60, DifCir, differential analysis of eccDNA; DExD/H‐Box helicase 60; DNASE1L3, deoxyribonuclease 1‐like 3; eccDNA, extrachromosomal circular DNA; ELISA, enzyme‐linked immunosorbent assay; GDF‐15, growth‐differentiation factor (GDF)‐15; HC, healthy controls; GDF‐15, growth differentiation factor 15; His, histidine; HPLC‐MS/MS, high performance liquid chromatography‐tandem mass spectrometry; IMN, idiopathic membranous nephropathy; IFI44L, IFN‐induced protein 44‐like; ICx, immune complexes; IFIT3, interferon‐induced protein with tetratricopeptide repeats 3; IFN‐ɑ, interferon‐alpha; IgM, immunoglobulin M; IFI44, interferon induced protein 44; j‐SLE, Juvenile systemic lupus erythematosus; MAP‐2, anti‐microtubule associated protein 2; MX1, GTPbinding protein Mx1; MINA, Myc‐induced nuclear antigen; MX2, MX dynamin like GTPase 2; NPV, negative predictive value; NPSLE, neuropsychiatric systemic lupus erythematosus; NRGs, NETs‐related genes, NETs, neutrophil extracellular traps; OAS3, 2′‐5′‐oligoadenylate synthase 3; S100A8, S100 calcium‐binding protein A8 protein; PHACTR4, phosphatase and actin regulator 4; P3H1, prolyl 3‐hydroxylase 1; PLA2R‐AB, phospholipase A2 receptor autoantibodies; pSLE, pediatric‐onset SLE; PBMC, peripheral blood mononuclear cell; PCR, polymerase chain reaction; qRT‐PCR, real‐time reverse transcription PCR; RT‐qPCR, reverse transcription quantitative polymerase chain reaction; RT‐PCR, reverse‐transcription PCR; PPV, positive predictive value; ROC, receiver operating characteristic; RGS12, regulator of G‐protein signaling 12; RGC‑32, response gene to complement‑32; RNARNA‐seq:‐seq, ribonucleic acid sequencing; SVM, support vector machine; SLE‐PAH, systemic lupus erythematosus‐associated pulmonary arterial hypertension; sTREM‐1, soluble triggering receptor expressed on myeloid cells‐1; sNfL, serum neurofilament light chain; STAT1, signal transducer and activator of transcription 1; SiMoA, single molecule array; Sema4A, Semaphorin 4A; SLEDAI, systemic lupus erythematosus disease activity index; sTREM‐1, soluble triggering receptors expressed on myeloid cell‐1; STAT2, signal transducer and activator of transcription 2; TOMM40, mitochondrialimport receptor subunit TOM40 homolog; tsRNAs, tRNA‐derived small noncoding RNA; Tie2, tyrosine kinase receptor; VSIG4, V‐set immunoglobulin domain‐containing protein 4; WB, whole blood; α‐Klotho, single‐pass transmembrane protein ɑ‐Klotho.

**TABLE 2:** Emerging activity markers of SLE.

Organ‐specific	Marker	Specimen	method	Key points	Reference
	CD137	PBMCs* serum	ELISA	Serum sCD137 level is positively correlated with the percentage of CD4+CD137+ cells, which can be used as a biomarker of disease activity.	[56]
	PD‐1*	PBMC	ELISA	The expressions of PD‐1, PD‐L1*, and sPD‐1* were negatively correlated with SLE disease activity and could be used as potential biomarkers for SLE.	[57]
	IFN*	serum	RT‐PCR	Serum IFN activity was significantly increased in SLE patients. There was a moderate positive correlation with the total score of EULAR/ACR‐2019.	[58]
	Anti‐dsDNA*	PBMCs serum	The SLE‐ELISpot assay	When SLE‐ELISpot activity critical value (≥11.24 points), the sensitivity was 57.1%, the specificity was 83.9%.	[59]
	Platelet LGALS3BP*	platelet	RNA‐seq	Platelet release levels of LGALS3BP are highly correlated with circulating LGALS3BP (associated with disease severity)	[60]
	PHACTR2*, GOT2*, SELL*, CMC4*, MAP2K1*, CMPK2*, ECPAS*, SRA1*, STAT2*	PBMC	ELISA	AUC = 0.990 (95% CI = 0.968–1)	[29]
	Anti‐GAPDH*	Serum	ELISA	It was positively correlated with SLEDAI‐2K, ESR, IgG and IgM.	[61]
	AGR*	Blood	Ordinal logistic regression analysis	AGR (*β* = −1.319, 95% confidence interval [CI] –2.595, –0.042; *p* = .043) is an independent risk factor for SLE disease activity.	[62]
	lncRNA panel (MIR31HG, NKILA)		qRT‐PCR	It was significantly correlated with albumin/creatinine ratio, glomerular filtration rate and SLEDAI score.	[63]
	AGEs*	Skin	Skin autofluorescence	The AGEs of SLE was 0.721, higher than that of healthy control group.	[64]
	T cell expressions of aberrant gene signatures and Co‐IRs*	PBMC	ELISA	It is associated with disease activity, nephritis, and response to treatment in patients with SLE.	[65]
	PLR*		Meta‐analyses	PLR (SMD = 0.604, 95% CI = 0.299–0.909, *p* = .001) in SLE patients was significantly higher than normal.	[66]
	gene EPSTI1	PBMCs	RT‐qPCR	In patients with SLE, EPSTI1 was positively associated with disease activity and T cell‐related genes.	[67]
LN	IGBP1*	Serum	ELISA	The specificity and sensitivity of serum IGBP1 level (critical value 547.45 ng/mL) were 96.9% and 93.8%, which can be used as biomarkers for active nephritis.	[68]
LN	CD56brightCD16− to CD57+CD56dimCD16+ NK cell ratio	Blood, serum	Flow cytometry	The AUC for patients with severe SLE was 0.722, with an optimal cut‐off of 0.075, and the ratio for patients with LN was 0.773, with an optimal cut‐off of 0.108.	[69]
LN	usCD163*	Blood, urine	ELISA	usCD163 levels were significantly elevated in patients with active LN and correlated with UPCR, disease activity, and anti‐DSDNA Ab levels.	[70]
LN	uNGAL*, uKIM*, uNGAL/Creat ratio, and uKIM/Creat ratio	Urine	ELISA	When uNGAL level is above 59, the specificity and sensitivity of diagnosis of active nephritis are 100%, 95%, AUC = 0.996. In addition, at levels above 1.6, its specificity is 80%, sensitivity is 95%, and AUC = 0.919.	[71]
LN	MX2	Whole blood and peripheral blood	qRT‐PCR	MX2 is highly expressed in SLE and positively correlated with SLE disease severity and SLEDAI.	[41]
LN	sALCAM*	Urine	ELISA	Correlation coefficients ranging from 0.35 to 0.41	[72]
LN	VCAM‐1*	Urine	ELISA	AUC: 0.81, *p* = .009, sensitivity and specificity vales ranging from 78% to 92%	[73]
LN	ALCAM*	Urine	ELISA	AUC 0.75, *p* = .0001, sensitivity and specificity vales ranging from 78% to 92%	[73]
LN	PF4*	Urine	ELISA	AUC 0.778, *p* = .001	[73]
LN	Ungal*, uKIM‐1*	Serum	ELISA	When uNGAL level is greater than 59, the sensitivity and specificity of LN detection are 95% and 100%. When the uNGAL/creatinine ratio is greater than 92, the sensitivity and specificity are 100% and 97%.	[74]
LN	Nrf2*	Blood	Kruskal–Wallis test	Serum Nrf2 protein levels in LN patients were positively correlated with kidney injury and SLEDAI.	[75]
LN	NLR*	Blood	Kruskal–Wallis test	The NLR specificity was 75%, the sensitivity was 71.9%, the AUC = 0.747.	[76]
LN	PLR*	Blood	Kruskal–Wallis test	The PLR sensitivity was 87.5%, the specificity was 50%, and the AUC was 0.658.	[76]
LN	SIRI*	Blood	Kruskal–Wallis test	SIRI has an AUC of 0.627, an optimal cut‐off of 1.225 (*p* = .081), a specificity of 62.5%, and a sensitivity of 65.6%.	[76]
LN	SII*	Blood	Kruskal–Wallis test	The AUC of SII was 0.708, the specificity was 0.750, and the sensitivity was 0.719.	[76]
ACLE*	TNF‐α*		Logistic regression analysis	AUC = 0.716	[77]
cSLE	Hemopexin	Urine	ELISA	AUC: 0.81, *p* < .0001	[73]
NPSLE	IL‐6*	CSF	Retrospectively analyzed	The IL‐6 level of CSF before treatment was 29.1 pg/mL, which was significantly decreased compared with 3.8 pg/mL after treatment (*p* = .008).	[78]
NPSLE	HMGB1	Serum	ELISA	A significant correlation between HMGB1 levels and SLEDAI‐2k (*r* = 0.6527, *p* = .000), AUC (95%CI): 0.843 (0.783–0.903) OR: 1.7	[79]

*Anti‐dsDNA, anti‐double‐stranded DNA; anti‐GAPDH, glyceraldehyde 3‐phosphate dehydrogenase autoantibodies; ACLE, Acute cutaneous lupus erythematosus; AGR, albumin‐to‐globulin ratio; AGEs, Advanced glycation end‐products; Co‐IRs, co‐inhibitory receptors; ALCAM, activated leukocyte CAM; CAM, cell adhesion molecule; CMC4, Cx9C motif‐containing protein 4; CMPK2, cytidine/uridine monophosphate kinase 2; ECPAS, Ecm29 proteasome adaptor and scaffold; GOT2, glutamate oxaloacetate transaminase 2; IGBP1, immunoglobulin‐binding protein 1; IL‐6, Interleukin‐6; IFN, interferon; LGALS3BP, soluble 3 binding protein; MAP2K1, dual specificity mitogen‐activated protein kinase kinase 1; Nrf2, nuclear factor erythroid 2‐related factor 2; NLR, neutrophil/lymphocyte ratio; PLR, the platelet‐to‐lymphocyte ratio; PLR, platelet/lymphocyte ratio; PF4, platelet factor‐4; PBMCs, peripheral blood mononuclear cells; PHACTR2, phosphatase and actin regulator 2; PD‐1, programmed death‐1; PD‐L1, programmed death ligand‐1; sALCAM, soluble activated leukocyte cell adhesion molecule; sPD‐1, the soluble form of PD‐1; SELL, lL‐selectin; SRA1, steroid receptor RNA activator 1; STAT2, transcription 2; SIRI, systemic inflammatory response index; SII, systemic immuneinflammatory index; TNF‐α, tumor necrosis factor‐alpha; usCD163, urine‐soluble CD163; uKIM, urinary kidney injury molecule; uNGAL, urinary neutrophil gelatinase‑associated lipocalin; uKIM‑1, urinary kidney injury molecule‑1; uNGAL, urinary neutrophil gelatinase‑associated lipocalin; uKIM‐1, kidney injury molecule‐1; VCAM‐1, vascular CAM‐1.

**TABLE 3:** Other potential SLE markers.

Organ‐specific	Marker	Specimen	Method	Key points	Reference
	GAPDH	Serum	ELISA	AU = 83.07 (43.66–115.8) in NPSLE group and AU = 68.46 (46.48–93.81) in non‐NPSLE group, *p* = .0588.	[61]
	miR‐101‐3p*	PBMC	ELISA	The level of miR‐101‐3p in PBMC cells in SLE was significantly decreased.	[80]
	m6A*	PBMC	RT‐qPCR	The alteration of m6A modification in SLE can affect the inflammatory process and participate in the pathogenesis of SLE.	[81]
	C3 level	Serum	ELISA	When the cut‐off value was ≥0.780 g/L, the specificity was 75.0% and the sensitivity was 70.6%, AUC = 0.756 (*p* = .003).	[82]
LN	SUA	Serum	Retrospective observational study	SUA in LN is associated with renal severity.	[83]
LN	Serum salusin‐β	Serum	ELISA	Salusin‐β in SLE group was 474.2 ± 117.1 pg/mL, which was significantly higher than that in healthy group (157.7 ± 88.7 pg/mL). (*p* = .001)	[84]
LN	NET* remnants (Elastase‐DNA and HMGB1‐DNA complexes)	Serum	ELISA	NET remnants is significantly increased in LN and can be used as a marker to identify poor prognosis.	[85]
LN	OPN*	Serum	ELISA	OPN in LN was higher than that in normal people or SLE patients with other organ involvement (*p* < .0001 and 0.0032, respectively), independent of renal activity, and correlated with low complement levels in LN.	[86]
NPSLE	AECA*	Serum	ELISA	AECA was found in 11 (64.7%) of 17 NPSEL patients and 10 (29.4%) of 34 non‐NP SLE patients	[87]
NPSLE	Autoantibodies to TPI*	Serum	ELISA	Ten of the 31 NPSLE patients had anti‐TPI positivity (32.3%), sensitivity 32.3%, specificity 95% for NPSLE	[88]
LE*	Anti‐gAChRα3 Abs*	Serum	Luciferase immunoprecipitation system assay.	The odds ratio of anti‐Gachrα3 antibody was 6.2 (95%CI: 1.9–20.3, *p* = .002), and it could be used as a predictor of LE.	[89]
Fatigue with SLE	NR2(NMDAR* subunit)	Serum	ELISA	Anti‐NR2 antibodies were detected in 46.9% of SLE patients with fatigue (titer > 2 ng/mL).	[90]
CVD	NT‐proBNP*	Serum	Multivariable logistic regression model	The AUC of NT‐proBNP = 0.78 (95%CI: 0.69–0.87), with a threshold of 133 pg/mL, was strongly correlated with CVD.	[91]

*Note*: Markers related to SLE severity, occurrence, development, occurrence or recurrence, and treatment.

* AECA, anti‐endothelial‐cell antibodies; anti‐gAChRα3 Abs, anti‐ganglionic nicotinic acetylcholine receptor α3 subunit (gAChRɑ3) antibodies (Abs); LE, Lupus enteritis; m6A, N6‐methyladenosine; miR‐101‐3p, microRNA‐101‐3p; NET, neutrophil extracellular trap; NT‐proBNP, N‐terminal pro‐brain natriuretic peptide; NMDAR, antiN‐methyl‐d‐aspartate receptor; OPN, osteopontin; SUA, serum uric acid; TPI, triosephosphate isomerase.

## 4 BIOMARKERS IN SLE

The manifestations of SLE are associated with a variety of autoantibodies, leading to the formation and deposition of immune complexes and other immune system processes. The clinical manifestations and pathogenesis of SLE are very complex, which brings challenges to understanding and definition.^92^ At present, as a complex immune disease, the pathogenesis and etiology of SLE are not yet clear, and there are few markers that can be used in clinical practice.^93^ SLE is diagnosed and classified according to the patient's clinical manifestations, immune and inflammation‐related biomarkers, and it is very important to develop a unified diagnostic criteria.

At present, the most commonly used diagnostic criteria in the world is the 2019 EULAR/ACR SLE classification standard based on SLICC‐2012 and ACR‐1997, which has better sensitivity and specificity.^94^

A hallmark of SLE is the dysregulation of autoreactive B cells and many other types of immune cells, including myeloid cells. There are autoreactive B cells in SLE. The B cell receptor on the surface of B cells can recognize certain DNA and RNA‐related antigens, which are derived from the increased abundance of neutrophil extracellular traps (NETs), antigens released by necrotic or apoptotic cells due to disordered processes, and initiate cell internalization. Once in the endosome, autoantigens can trigger Toll‐like receptor 7 (TLR7) and/or TLR9 in B cells. TLR7 drives two different pathways: germinal center response and extrafollicular pathway, to produce pathogenic antibody‐secreting cells, which are involved in autoantibody production and disease pathogenesis. Genetic or environmental signals lead to overexpression of TLR7, which increases the susceptibility of individuals to SLE. Under physiological conditions, TLR9 leads to the loss of tolerance of B cells to autoantigens by limiting the stimulating activity of TLR7. The intracellular protein UNC93B1 drives TLR7 and TLR9 to transport to the endosomal compartments. The disruption of TLR9 function can lead to a D34A mutation in transmembrane UNC93B1, which is conducive to its interaction with TLR7, Increased abundance and signal transduction of TLR7 in endosomes, and triggering a fatal systemic inflammatory syndrome.^95^

The pathogenesis of SLE involves a variety of pathogenic mechanisms. Mitochondrial dysfunction, lipid metabolism disorders, and mTOR signaling abnormalities play an important role in it. NET level increased; the loss of tolerance of B cells to autoantigens and T cell dysfunction caused by mitochondrial hyperpolarization, reactive oxygen intermediates, and decreased ATP levels activate innate and adaptive immunity. Studies have shown that these abnormal activation pathways will be potential therapeutic targets for systemic lupus erythematosus treatment.^96^, ^97^ Lipid metabolism disorders increase the risk of disease onset, lipid accumulation, inflammation and cardiovascular disease in SLE patients through atherosclerosis.^98^ CVD is the main cause of death in patients. Intervention of metabolic pathways through treatment may be a promising strategy for controlling atherosclerosis and inflammation in systemic lupus erythematosus. In addition, proteins, peptides, and amino acids are important substrates involved in immune cell metabolism. These substrates are dependent on the activation of the serine‐threonine protein kinase mTOR, which is essential for maintaining immune cell homeostasis. Increased immune cell differentiation and proliferation, pro‐inflammatory cytokine secretion, and ROS production have been shown to be associated with abnormalities in the mTOR pathway in SLE patients.^99^ The mTOR signaling pathway is closely related to the pathogenesis of SLE. Targeting mTOR may also be an effective treatment for clinical SLE management.

### 4.1 Biomarkers in lupus nephritis (LN)

Lupus nephritis is the most serious and important manifestation of SLE. It usually occurs within 5 years after diagnosis, and a small number of people can develop into end‐stage within 10 years.^100^, ^101^, ^102^, ^103^

Because of the risk of invasive renal biopsy, a biopsy is not widely used in all health services; it requires infrastructure and training, and there is a risk of complications, such as hematuria, kidney damage and loss, and even death. Therefore, renal biopsy is not a routine monitoring method for LN.^104^ Therefore, renal biopsy is not a daily monitoring method for LN. We need more noninvasive and convenient means, namely biomarkers, to diagnose diseases or predict the occurrence and development of diseases.

#### 4.1.1 Plasma biomarkers

Plasma C4d, The final cleavage product of C4 during complement activation is a valuable marker to identify the high activity of LN disease.^105^ Studies on C3dg and membrane attack complex (MAC) by Shi et al. showed that plasma C3dg and MAC depositions may be potential biomarkers of LN disease severity and tissue damage. C3dg and MAC deposition can be used in parallel as independent predictors, and the specificity of the model is the highest at 75%. It can also be simultaneously deposited (in series) as an independent predictor, and the sensitivity of the model is the highest at 100%. MAC and C3dg staining may contribute to the routine study of lupus biopsy to better help treatment.^106^

Tumor necrosis factor receptor I (TNF‐RI) is a classical ligand of tumor necrosis factor‐α (TNF‐α), which is widely expressed in the human body.^107^ It can activate the signal pathway of cell death and promote the expression of inflammation, which is closely related to the pathogenesis of SLE patients.^108^, ^109^ The research data of Liu et al. showed that TNF‐RI has high specificity and sensitivity in the identification of LN and SLE patients, and can be used as an ideal clinical tool. Plasma sTNF‐RI may be an effective marker for detecting LN disease activity.^107^

#### 4.1.2 Serum biomarkers

Interleukin‐23 (IL‐23) is a pro‐inflammatory cytokine that promotes the expression of inflammation. It is closely related to the pathogenesis and disease activity of SLE. It can be an effective marker of SLE and a therapeutic target in the future.^110^

IL‐32 is a recently introduced pro‐inflammatory cytokine that is involved in various autoimmune diseases. IL‐32 is mainly expressed in glomerular lesions, suggesting that it is related to the deposition of immune complexes leading to LN.^111^

β2‐microglobulin is a protein that is linked to the light chain of major histocompatibility complex class I antigen. The study of Dalia Mohamed Gamal et al. showed that the sensitivity of serum b2MG in predicting LN, disease activity, and cumulative damage was as high as 90%, and both have high specificity, positive predictive value, and negative predictive value. Therefore, serum b2MG levels will become a valuable predictor of renal involvement, clinical disease activity, and injury scores in SLE patients.^112^

In juvenile SLE (JSLE), serum Cystatin C (Cys C) is associated with routine indicators of renal impairment and disease activity index (AI). Serum Cys C level may be a promising noninvasive biomarker for predicting renal disease activity and biopsy grade in children with JSLE.^113^

ROC analysis by Reshetnyak et al. showed that the AUC of the myeloperoxidase‐ deoxyribonucleic acid (MPO‐DNA) complex was about 0.87. When the cut‐off value of MPO‐DNA complex was 0.058, the sensitivity was 80%, and the specificity was 74.5%. In SLE patients, the level of MPO‐DNA complex will be a promising marker for renal involvement, clinical disease activity, and immune metabolic disorders.^114^

Tang et al. found that serum V‐set immunoglobulin domain‐containing protein 4 (VSIG4) level was longitudinally tracked with LN disease activity, which could reflect the renal pathological AI, especially the AI components of crescent formation and transparent deposits. VSIG4 will become an effective biomarker for the diagnosis of LN, monitoring the activity of LN, and a therapeutic target in the future.^115^

Salusin‐β can also be used as a potential biomarker for SLE nephritis and thrombosis. Hajialilo et al. detected that serum salusin‐β levels in the SLE group and control group were significantly different. Multivariate linear regression analysis showed that serum salusin‐β level was still significantly correlated with nephritis and thrombosis after adjustment for the model of serositis, nephritis, and thrombosis. Salusin‐β may play a role in the pathogenesis of SLE.^116^

#### 4.1.3 Urine biomarkers

Interferon‐induced galectin‐3 binding protein (Gal‐3BP) is a secreted scavenger protein belonging to the lectin family.^117^, ^118^, ^119^ Urinary Gal‐3BP (u‐Gal‐3BP) is a potential noninvasive marker for monitoring renal activity in LN patients.^120^ The level of CD163 was significantly correlated with SLE Disease AI, renal SLEDAI, urinary protein excretion, and complement C3 level. Urinary CD163 is expected to be a marker for the identification of active renal disease in children with SLE.^121^

Small RNAs (tsRNAs), derived from mature tRNAs or their precursors, are a new type of noncoding small RNAs (14–40 nt) that are produced under enzymatic hydrolysis and stress conditions and are stable in exosomes. Including tRNA‐derived fragments (tRFs) and half‐molecules (tiRNAs).^122^ The signaling pathway analysis of tiRNA5‐Lys‐CTT‐1 showed that tsRNA was closely related to the pathogenesis of lupus by participating in human papillomavirus infection, tumor senescence and autophagy, EGF/EGFR signaling pathway, and other pathways, and urine exosomal tsRNAs can be used as a noninvasive biomarker for effective diagnosis and prediction of SLE nephritis.^123^

Yuan et al. found that the increase of urinary modified C‐reactive protein (mCRP) level could predict tubulointerstitial lesions, and its ROC‐AUC was 0.766. Urinary mCRP levels were significantly increased in LN patients. The sensitivity and specificity of detecting urinary mCRP level to identify tubulointerstitial lesions were 79.3% and 62.5%, respectively, so it may be used as a biomarker of renal tubulointerstitial lesions in LN patients.^124^

The levels of proteinuria and urine (u)‐kidney injury molecule‐1 (u‐KIM‐1) in active LN patients were higher than those in inactive LN patients. With the strengthening of treatment, the levels of proteinuria and u‐KIM‐1 decreased, and the percentage of glomerular crescent formation was related. The level of u‐KIM‐1 was related to LN disease activity and renal pathological manifestations, so it could predict the treatment response.^125^

Urine soluble triggering receptor expressed on myeloid cells‐1 (sTREM‐1) is a potential marker for clinical use, which is different from plasma sTREM‐1. Molad et al. reported that the level of sTREM‐1 was higher in the whole SLE cohort than in the healthy control group, and higher in the active renal lupus group than in other groups. Urine sTREM‐1 level was correlated with renal SLEDAI score, serum C3, C4 levels, and proteinuria, while plasma sTREM‐1 level was not correlated with renal SLEDAI score, which could be used as a potential biomarker for active renal SLE.^126^

Urinary long noncoding RNA (lncRNA) may also help to identify and predict the histological type of LN. Szeto et al. found some urinary lncRNA targets. Among them, the level of urinary cancer susceptibility candidate 2 (CASC2) can evaluate the histological grade of nephritis, which may be the highest in patients with simple grade V nephritis. The level of urinary metastasis‐associated lung carcinoma transcript 1 (MALAT1) was significantly correlated with histological AI. When the cutoff value was 12, the diagnostic sensitivity of MALAT was 96.2%. When the cutoff value was 13, the diagnostic sensitivity of CASC2 was 70.5%. The specificity of both was 100%. Their characteristics indicate that they may affect the phenotype of lupus nephritis and can be used to determine the histological classification of LN in clinical practice. At the same time, urine taurine upregulated gene 1 (TUG1) may be related to the generation of glomerular permeability barrier damage and the regulation of immune complex deposition. The sensitivity of the TUG1 level for diagnosis was 79.2%, and the specificity was 75.0%.^127^

#### 4.1.4 Other biomarkers for LN

Patients with positive peritubular capillary C4d (PTC‐C4d) have a higher proportion of type IV LN, which is significantly different from patients with negative PTC‐C4d, and the disease activity or severity of adult patients with LN is less than that of children. PTC‐C4d staining can be used as a more sensitive biomarker for pediatric LN.^128^ Through data set analysis, interferon‐inducible protein 16 (IFI16) showed strong positive expression in the LN group.^129^ Renal IFI16 can be used as a marker for monitoring disease activity and evaluating prognosis in LN patients. Through weighted gene co‐expression network analysis, four lncRNAs associated with disease activity were selected as core genes to participate in the key biological processes of LN disease activity, which were NRIR, KLHDC7B‐DT, MIR600 HG, and FAM30 A, respectively. The first two play a key role in regulating interferon (IFN)‐mediated processes. FAM30A and MIR600HG may play an important role in B cell regulation in LN by influencing immunoglobulin gene expression and IFN‐λ pathway through cis‐regulatory and competitive endogenous RNA mechanisms.^130^ Therefore, they are very promising blood biomarkers and LN liquid biopsy.

### 4.2 Biomarkers for neuropsychiatric in SLE (NPSLE)

NPSLE is another important complication of SLE, which can affect the central and peripheral nervous system and is the second leading cause of death and morbidity in SLE patients.^131^ The accurate prevalence of NPSLE has not been investigated, but the proportion of NPSLE in SLE patients is estimated to be between 12% and 95% by published data.^132^, ^133^, ^134^, ^135^ There are currently some biomarkers that can accurately identify NPSLE and exclude non‐neuropsychiatric SLE, such as serum interleukin (IL)‐6,^79^ cerebrospinal fluid (CSF) IL‐6^136^ and immunoglobulin (Ig) G and/or IgM isotypes serum anti‐β2‐glycoprotein I (β2GPI) antibodies.^137^ Biomarkers for NPSLE can be obtained from serum or CSF. Compared with patients without neuropsychiatric manifestations, serum neurofilament light chain (sNfL) levels were significantly increased in NPSLE.^138^ Serum anti‐ribosomal P antibody was also correlated with NPSLE.^139^, ^140^ The study found that the AUC of CSF α‐Klotho was 0.94,^141^ and the decrease of α‐Klotho protein was related to endothelial dysfunction and neuronal damage.^142^, ^143^ It has been found that serum high mobility group box protein 1 levels were elevated in NPSLE patients and were positively correlated with disease activity.^144^ The levels of pro‐inflammatory cytokines and chemokines in CSF of NPSLE patients will increase, including IL‐6, IL‐8, IL‐10, TNF‐α, interferon‐gamma, C‐C motif ligand 2/monocyte chemoattractant protein‐1, C‐X‐C motif ligand 10/inducible protein‐10 (IP‐10) and other cytokines.^145^, ^146^ They are potential biomarkers for judging the diagnosis of NPSLE or the occurrence and development of disease activity, and have certain research value and clinical application prospects.

### 4.3 Biomarkers for skin lesions in SLE

Among the other organs affected by SLE, the skin ranks second, and 80% of SLE patients will have skin manifestations during the course of the disease.^147^ It has been reported that 5%–25% of patients with cutaneous lupus erythematosus (CLE) progress to SLE during the course of the disease.^148^, ^149^, ^150^ When progressing from CLE to SLE, patients also experience extracutaneous symptoms such as joint pain, arthritis, and other musculoskeletal symptoms in the proximal interphalangeal and knee joints.^151^, ^152^ The primary feature of systemic involvement in CLE patients is the presence of antinuclear antibodies (ANA).^153^ Elevated ANA, anti‐ds DNA and anti‐Sm antibody titers are recognized as important indicators of major system involvement and disease progression to SLE in CLE patients. In addition, leucopenia, thrombocytopenia, anemia and high ESRs can also be used as indicators of progression to SLE.^154^ Common autoantibodies in patients with cutaneous SLE include anti‐ribosomal P protein and anti‐galactose lectin 3. Ro52 (also known as TRIM21) antibodies have also been found. In mice, the absence of Ro52 induces lupus‐like skin diseases.^155^ Many studies have found that the level of IFN‐α and the expression of IFN‐λ1 in the skin lesions of CLE and SLE patients and the serum of SLE patients are increased.^156^, ^157^, ^158^, ^159^

### 4.4 Biomarkers for cardiovascular involvement in SLE

Cardiovascular disease (CVD) is one of the most important complications of SLE, and is an important factor leading to the onset and death of SLE patients.^160^ A major manifestation of CVD is atherosclerosis, and an important mechanism in the pathogenesis of atherosclerosis is endothelial dysfunction, which is characterized by the loss of vasodilation and inflammation and prothrombotic state and can predict future clinical CV events.^161^, ^162^ In addition to promoting the secretion of IFN‐α by plasmacytoid dendritic cells, CD4 + CXCR3 + T cells are also related to the increased size of atherosclerotic plaques under stimulation.^163^ It has been reported that the level of serum cell adhesion molecules in SLE patients is significantly elevated compared with healthy controls, which is a biomarker for monitoring disease activity.^164^, ^165^

## 5 CONCLUSIONS

For SLE patients, the key to good efficacy and prognosis lies in accurate diagnosis, close monitoring, and early treatment. Reliable SLE biomarkers can provide information at different time points in the disease process, such as accurate diagnosis and early treatment from the inflammatory stage of the disease, the evaluation of terminal organ damage, or the evaluation of treatment response, which can significantly improve the therapeutic effect and prognosis of SLE. However, due to the complex pathogenesis and clinical heterogeneity of SLE, it is difficult to identify and develop SLE‐specific biomarkers, so any single biomarker will not become a “lupus biomarker.” In the face of these challenges, the use of various omics techniques, meta‐analysis, mathematical models combined with multiple biomarkers, and advanced computational methods to analyze large data sets may be a good idea for evaluating and discovering new biomarkers SLE.

## AUTHOR CONTRIBUTIONS

**Su‐jie Zhang:** Conceptualization (lead); writing—review and editing (equal); writing—original draft (lead). Rui‐yang Xu: Conceptualization (supporting); writing—original draft (supporting). **Long‐li Kang:** Funding acquisition; project administration; supervision; writing—review and editing (equal).

## CONFLICT OF INTEREST STATEMENT

The authors declare no conflict of interest.

## ETHICS STATEMENT

All the data in this study came from authoritative databases, and no human or animal experiments were conducted. Therefore, it does not require the approval of the Ethics Committee.

## Supporting information

Supporting information.

Supporting information.
