# Supplementary material for: Biomarkers for systemic lupus erythematosus: A scoping review
Source: Immun Inflamm Dis. 2024 Oct 4;12(10):e70022. doi: 10.1002/iid3.70022 (PMC11450456; doi:10.1002/iid3.70022)
Supplement: Supplementary file 1 — Supporting information. [file IID3-12-e70022-s002.docx]

TABLE 1,2,3

**TABLE 1**| Potential diagnostic markers for SLE.

| **Organ-**  **Specific** | **Marker** | **Specimen** | **method** | **Key Points** | **Reference** |
| --- | --- | --- | --- | --- | --- |
|  | Circulating Exosomal microRNAs | serum | PCR* | AUC* of 0.790 for exosomal miR21,  AUC of 0.709 for exosomal miR-155. | ^16^ |
|  | DDX60* | blood | RT-qPCR* | The AUC for predicting high disease activity of SLE was 0.8818. | ^17^ |
|  | serum leptin | serum | ELISA* | AUC = 0.972, when the cut-off value was 9.9 ng/mL, the accuracy, specificity, sensitivity, NPV* and PPV* of leptin were 90.8%, 92%, 90%, 86.8% and 94%, respectively. | ^18^ |
|  | adiponectin | serum | ELISA | AUC = 0.833, when the cut-off value was 9.4 ng/mL, the accuracy of adiponectin was 80.8%, the specificity was 82%, the sensitivity was 80%, the NPV was 74.5%, and the PPV was 86.2% . | ^18^ |
|  | Anaerococcus, Gardnerella, Lactobacillus | feces | PCR | The probability of difference between SLE and HC* among Anaerococcus, Gardnerella and Lactobacillus was 82.80 ~ 83.60%. | ^19^ |
|  | Bacteroides, Escherichia-Shigella, Streptococcus | vagina | PCR | The accuracy of the identification of Bacteroides, Escherichia-Shigella, and Streptococcus between SLE patients and HC patients was 89.90%−100.00%. | ^19^ |
|  | HMGB1(NRGs*) | WB*,  PBMC* | the correlation and protein-protein interaction analyses | AUCs of 0.930 for HMGB1,showed its potency as useful diagnostic biomarkers | ^20^ |
|  | ITGB2(NRGs) | WB,  PBMC | the correlation and protein-protein interaction analyses | AUCs of 0.901 for ITGB2,showed its potency as useful diagnostic biomarkers | ^20^ |
|  | CREB5(NRGs) | WB,  PBMC | the correlation and protein-protein interaction analyses | AUCs of 0.788 for CREB5, CREB5 is involved in PI3K-Akt and Toll-like receptor signaling pathways leading to SLE. | ^20^ |
|  | IgA* | saliva | ELISA | The salivary IgA subtype is associated with disease, with an AUC of 0.855 for IgA1 and 0.761 for IgA2. | ^21^ |
|  | miR-342-3p | serum | qRT-PCR* | The expression of miR-342-3p in SLE patients was significantly lower than that in healthy people. | ^22^ |
|  | Sema4A* | serum | ELISA | Sema4A is positively correlated with SLEDAI and has high diagnostic value for SLE. | ^23^ |
|  | sTREM-1* | serum | PCR,ELISA | Serum sTREM-1 was significantly elevated in SLE patients, with AUC= 0.9511. | ^24^ |
|  | IFI44L* | PBMC | RT-qPCR | ROC* analysis showed that IFI44L had diagnostic significance for SLE. | ^25^ |
|  | RGC‑32* | serum | ELISA | AUC=0.803, when RGC-32≥206.4 pg/mL, the specificity and sensitivity of SLE diagnosis were 85% and 77.5% . | ^26^ |
|  | His* | plasma | RNA-seq* | Data analysis showed that His could effectively identify SLE. | ^27^ |
|  | cf-eccDNA* | blood | DifCir* | The number of eccDNA in the healthy control group was lower than that in DNASE1L3* deficient SLE patients, and the eccDNA was reduced by 0.0321 times. | ^28^ |
|  | IFIT3*\MX1*\  TOMM40*\STAT1*\  STAT2*\OAS3* | PBMC | ELISA | AUC = 0.723（95% CI = 0.591–0.854） | ^29^ |
|  | S100A8* | serum | ELISA | AUC=0.831 (95% CI, 0.765–0.897)Sensitivity :61%Specificity:91.1%  PPV:95.7% | ^30^ |
|  | S100A8 | urine | ELISA | AUC=0.751 (95% CI, 0.648–0.854)Sensitivity:99%Specificity:55.6%  PPV:63.9% | ^30^ |
|  | S100A8 | salivary | ELISA | AUC=0.729 (95% CI, 0.646–0.812).Sensitivity:52%Specificity:91.1%  PPV:87.3% | ^30^ |
|  | ABCB1 | PBMC | qRT-PCR* | AUC=0.754 | ^31^ |
|  | IFI27 | PBMC | qRT-PCR | the diagnostic effect and discovered that the AUC values of the biomarkers, IFI27 were 0.875，p=0.2746 | ^31^ |
|  | PLSCR1 | PBMC | qRT-PCR | the diagnostic effect and discovered that the AUC values of the biomarkers, PLSCR1were 0.851，p=0.1376 | ^31^ |
|  | lncRNA SNHG1 | PBMCs | RT-qPCR | SNHG1 expression was positively correlated with SLEDAI score, IgG, CRP, and ESR, and negatively correlated with C3 and C4. | ^32^ |
|  | KLRB1, KLRF1, GZMK, IL-7R and CD40LG | PBMC | DEGs analysis | SVM* and LASSO regression analysis showed that KLRB1, KLRF1, GZMK, IL-7R and CD40LG had good diagnostic ability. | ^33^ |
|  | MINA* 53 protein | blood | ELISA，real-time PCR | When Mina53 serum level was 125.4, AUC=0.951 and 8.5, AUC=0.88, the sensitivity and specificity of SLE diagnosis were the highest. | ^34^ |
| LN | urinary exosome tsRNAs* | Urine | RT-PCR | tRF3-Ile-AAT-1: AUC = 0.777 ( specificity 66.69%, sensitivity 79.63%)  TirNA5-LYS-CT-1: AUC = 0.715 (specificity 76.92%, sensitivity 66.96%) | ^35^ |
| LN | Urine sTREM-1* | Blood,urine | ELISA | Urinary sTREM-1 level in SLE was higher than that in healthy group, and was positively correlated with renal sledai score, negatively correlated with serum C3 and C4 levels, and positively correlated with albuminuria. | ^36^ |
| LN | VSIG4* | serum | quantitative protein microarray | AUC of 0.93 for VSIG4. | ^37^ |
| LN | EGFR, FOLR2, PDGF-RB, and TFRC | renal biopsy | scRNA sequence data analysis and immunohistochemistry | EGFR, FOLR2, PDGF-RB, and TFRC have the potential to be novel LN biomarkers, but the expression profiles have not been confirmed. | ^38^ |
| LN | BCDF* | serum | ELISA | increased levels of BCDF in SLE patientsSensitivity80.6%，Specificity70.8% | ^39^ |
| LN | IgM* | serum | ELISA | increased levels of IgM in SLE patients Sensitivity 97.2%，Specificity 87.5% | ^39^ |
| LN | GDF-15* | serum | ELISA | GDF-15 was related to SLE pathogenesis,Sensitivity0.907,Specificity0.800 | ^40^ |
| LN | MX2* | whole blood and peripheral blood | qRT-PCR | The ROC curve for diagnostic efficacy validation of MX2 with AUCs of 0.958(GSE121239) and 0.9769(GSE11907) | ^41^ |
| LN | IFI44* | PBMC | qRT-PCR | The AUC of IFI44 was 0.850, the diagnostic specificity was 0.850, and the sensitivity was 0.923. | ^42^ |
| LN | Adiponectin | urine | ELISA | (18000 pg/ml)Sensitivity：91.7 %(95% CI)Specificity: 90.9% (95% CI), PPV52.4% (95% CI) | ^43^ |
| LN | MCP-1 | urine | ELISA | (1341 pg/ml)Sensitivity：37.5% (95% CI)Specificity：97.3% (95% CI)PPV：60.0% (95% CI) | ^43^ |
| LN | sVCAM-1 | urine | ELISA | (46000 pg/ml)and(103700 pg/ml)Sensitivity :79.2%and66.7%(95% CI)Specificity81.1%and95.5% (95% CI)PPV31.2%and31.2% (95% CI) | ^43^ |
| LN | PF4 | urine | ELISA | (134 pg/ml)Sensitivity ：83.3% (95% CI)  Specificity：93.7% (95% CI) PPV ：58.8% (95% CI) | ^43^ |
| LN | PHACTR4* | serum | ELISA | AUC:0.99 ,PHACTR4 ICx* were significantly elevated | ^44^ |
| LN | P3H1* | serum | ELISA | AUC: 0.82, P3H1 ICx was found significantly downregulated in LN | ^44^ |
| LN | RGS12* | serum | ELISA | AUC:0.90,RGS12 ICx was found upregulated in LN | ^44^ |
| j-NPSLE | neopterin | CSF* | liquid chromatography | Neopterin levels were significantly elevated in both active and inactive NPSLE patients. | ^45^ |
| j-NPSLE | IFN-α* | CSF | ELISA | It was significantly increased in both active and inactive NPSLE patients. | ^45^ |
| JSLE* | hsa_circ_0008945 | PBMCs | RT-qPCR | AUC=0.790 (95%CI: 0.6733-0.9067, P < 0.001), specificity 83.33%, sensitivity 70% | ^46^ |
| pSLE* | Ang*-1, Ang-2, and Tie2 | Serum  urine | ELISA | The AUC values of Ang-1, Ang-2 and Tie2 in serum and urine were all greater than 0.7. | ^47^ |
| cSLE | IFI44L* promoter methylation | blood | HRM-qPCR | AUC=0.867, specificity 1.000, sensitivity 0.753 . | ^48^ |
| NPSLE* | sNfL* | serum | SiMoA* | AUC =0.646 (95% CI: 0.554-0.738, p=0.003) | ^49^ |
| NPSLE | α-Klotho* | CSF | ELISA | AUC = 0.94 (p < 0.001) | ^50^ |
| NPSLE | L-Selectin* | CSF | 1000-plexed proteins array | sensitivity: 62.16%; specificity: 72.22% | ^51^ |
| NPSLE | Trappin-2 | CSF | 1000-plexed proteins array | sensitivity: 89.19%; specificity: 66.67% | ^51^ |
| NPSLE | TCN2 | CSF | 1000-plexed proteins array | AUC=0.703, P=0.0155 and sensitivity: 54.05%; specificity: 88.89% | ^51^ |
| NPSLE | CST6 | CSF | 1000-plexed proteins array | AUC=0.738, P=0.0043 and sensitivity: 51.35%; specificity: 94.44% | ^51^ |
| NPSLE | MAP-2* | CSF | ELISA | The positive rate of anti-MAP-2 antibody in NPSLE was 33.3% (8/24), the positive rate of CSF showed high specificity for NPSLE. | ^52^ |
| SLE‐PAH* | GDF‐15* | serum | enzyme‐linked immunosorbent assay | AUC=0.84, when GDF-15 ≥733 pg/mL, the specificity and sensitivity of SLE-PAH diagnosis were 58.6% and 91%. | ^53^ |
| IMN* | PLA2R-AB* | serum | ELISA | ROC curves of the PLA2R-AB showed that AUC: 0.907, Sensitivity 94.3%, Specificity 82.1%. | ^54^ |
| CVD* | Lactosylceramides | plasma | HPLC-MS/MS* | Lactosylceramides Correlate Negatively With Plaque Area and C3 | ^55^ |
| **Organ-**  **Specific** | **Marker** | **Specimen** | **method** | **Key Points** | **Reference** |
|  | Circulating Exosomal microRNAs | serum | PCR* | AUC* of 0.790 for exosomal miR21,  AUC of 0.709 for exosomal miR-155. | ^16^ |
|  | DDX60* | blood | RT-qPCR* | The AUC for predicting high disease activity of SLE was 0.8818. | ^17^ |
|  | serum leptin | serum | ELISA* | AUC = 0.972, when the cut-off value was 9.9 ng/mL, the accuracy, specificity, sensitivity, NPV* and PPV* of leptin were 90.8%, 92%, 90%, 86.8% and 94%, respectively. | ^18^ |
|  | adiponectin | serum | ELISA | AUC = 0.833, when the cut-off value was 9.4 ng/mL, the accuracy of adiponectin was 80.8%, the specificity was 82%, the sensitivity was 80%, the NPV was 74.5%, and the PPV was 86.2% . | ^18^ |
|  | Anaerococcus, Gardnerella, Lactobacillus | feces | PCR | The probability of difference between SLE and HC* among Anaerococcus, Gardnerella and Lactobacillus was 82.80 ~ 83.60%. | ^19^ |
|  | Bacteroides, Escherichia-Shigella, Streptococcus | vagina | PCR | The accuracy of the identification of Bacteroides, Escherichia-Shigella, and Streptococcus between SLE patients and HC patients was 89.90%−100.00%. | ^19^ |
|  | HMGB1(NRGs*) | WB*,  PBMC* | the correlation and protein-protein interaction analyses | AUCs of 0.930 for HMGB1,showed its potency as useful diagnostic biomarkers | ^20^ |
|  | ITGB2(NRGs) | WB,  PBMC | the correlation and protein-protein interaction analyses | AUCs of 0.901 for ITGB2,showed its potency as useful diagnostic biomarkers | ^20^ |
|  | CREB5(NRGs) | WB,  PBMC | the correlation and protein-protein interaction analyses | AUCs of 0.788 for CREB5, CREB5 is involved in PI3K-Akt and Toll-like receptor signaling pathways leading to SLE. | ^20^ |
|  | IgA* | saliva | ELISA | The salivary IgA subtype is associated with disease, with an AUC of 0.855 for IgA1 and 0.761 for IgA2. | ^21^ |
|  | miR-342-3p | serum | qRT-PCR* | The expression of miR-342-3p in SLE patients was significantly lower than that in healthy people. | ^22^ |
|  | Sema4A* | serum | ELISA | Sema4A is positively correlated with SLEDAI and has high diagnostic value for SLE. | ^23^ |
|  | sTREM-1* | serum | PCR,ELISA | Serum sTREM-1 was significantly elevated in SLE patients, with AUC= 0.9511. | ^24^ |
|  | IFI44L* | PBMC | RT-qPCR | ROC* analysis showed that IFI44L had diagnostic significance for SLE. | ^25^ |
|  | RGC‑32* | serum | ELISA | AUC=0.803, when RGC-32≥206.4 pg/mL, the specificity and sensitivity of SLE diagnosis were 85% and 77.5% . | ^26^ |
|  | His* | plasma | RNA-seq* | Data analysis showed that His could effectively identify SLE. | ^27^ |
|  | cf-eccDNA* | blood | DifCir* | The number of eccDNA in the healthy control group was lower than that in DNASE1L3* deficient SLE patients, and the eccDNA was reduced by 0.0321 times. | ^28^ |
|  | IFIT3*\MX1*\  TOMM40*\STAT1*\  STAT2*\OAS3* | PBMC | ELISA | AUC = 0.723（95% CI = 0.591–0.854） | ^29^ |
|  | S100A8* | serum | ELISA | AUC=0.831 (95% CI, 0.765–0.897)Sensitivity :61%Specificity:91.1%  PPV:95.7% | ^30^ |
|  | S100A8 | urine | ELISA | AUC=0.751 (95% CI, 0.648–0.854)Sensitivity:99%Specificity:55.6%  PPV:63.9% | ^30^ |
|  | S100A8 | salivary | ELISA | AUC=0.729 (95% CI, 0.646–0.812).Sensitivity:52%Specificity:91.1%  PPV:87.3% | ^30^ |
|  | ABCB1 | PBMC | qRT-PCR* | AUC=0.754 | ^31^ |
|  | IFI27 | PBMC | qRT-PCR | the diagnostic effect and discovered that the AUC values of the biomarkers, IFI27 were 0.875，p=0.2746 | ^31^ |
|  | PLSCR1 | PBMC | qRT-PCR | the diagnostic effect and discovered that the AUC values of the biomarkers, PLSCR1were 0.851，p=0.1376 | ^31^ |
|  | lncRNA SNHG1 | PBMCs | RT-qPCR | SNHG1 expression was positively correlated with SLEDAI score, IgG, CRP, and ESR, and negatively correlated with C3 and C4. | ^32^ |
|  | KLRB1, KLRF1, GZMK, IL-7R and CD40LG | PBMC | DEGs analysis | SVM* and LASSO regression analysis showed that KLRB1, KLRF1, GZMK, IL-7R and CD40LG had good diagnostic ability. | ^33^ |
|  | MINA* 53 protein | blood | ELISA，real-time PCR | When Mina53 serum level was 125.4(AUC=0.951) and Mina53 expression level was 8.5 (AUC=0.88), the sensitivity and specificity of SLE diagnosis were the highest. | ^34^ |
| LN | urinary exosome tsRNAs* | Urine | RT-PCR | The AUC of tRF3-Ile-AAT-1 was 0.777 (95% CI: 0.681-0.874, specificity 66.69%, sensitivity 79.63%), and that of TirNA5-LYS-CT-1 was 0.715 (95% CI: 0.715). 0.610-0.820, specificity 76.92%, sensitivity 66.96%), which can be used to distinguish SLE patients with LN from those without LN. | ^35^ |
| LN | Urine sTREM-1* | Blood,urine | ELISA | Urinary sTREM-1 level in SLE was higher than that in healthy group, and was positively correlated with renal sledai score, negatively correlated with serum C3 and C4 levels, and positively correlated with albuminuria. | ^36^ |
| LN | VSIG4* | serum | quantitative protein microarray | AUC of 0.93 for VSIG4. | ^37^ |
| LN | EGFR, FOLR2, PDGF-RB, and TFRC | renal biopsy | scRNA sequence data analysis and immunohistochemistry | EGFR, FOLR2, PDGF-RB, and TFRC have the potential to be novel LN biomarkers, but the expression profiles have not been confirmed. | ^38^ |
| LN | BCDF* | serum | ELISA | increased levels of BCDF in SLE patientsSensitivity80.6%，Specificity70.8% | ^39^ |
| LN | IgM* | serum | ELISA | increased levels of IgM in SLE patients Sensitivity 97.2%，Specificity 87.5% | ^39^ |
| LN | GDF-15* | serum | ELISA | GDF-15 was related to SLE pathogenesis,Sensitivity0.907,Specificity0.800 | ^40^ |
| LN | MX2* | whole blood and peripheral blood | qRT-PCR | The ROC curve for diagnostic efficacy validation of MX2 with AUCs of 0.958(GSE121239) and 0.9769(GSE11907) | ^41^ |
| LN | IFI44* | PBMC | qRT-PCR | The AUC of IFI44 was 0.850, the diagnostic specificity was 0.850, and the sensitivity was 0.923. | ^42^ |
| LN | Adiponectin | urine | ELISA | (18000 pg/ml)Sensitivity：91.7 %(95% CI)Specificity: 90.9% (95% CI), PPV52.4% (95% CI) | ^43^ |
| LN | MCP-1 | urine | ELISA | (1341 pg/ml)Sensitivity：37.5% (95% CI)Specificity：97.3% (95% CI)PPV：60.0% (95% CI) | ^43^ |
| LN | sVCAM-1 | urine | ELISA | (46000 pg/ml)and(103700 pg/ml)Sensitivity :79.2%and66.7%(95% CI)Specificity81.1%and95.5% (95% CI)PPV31.2%and31.2% (95% CI) | ^43^ |
| LN | PF4 | urine | ELISA | (134 pg/ml)Sensitivity ：83.3% (95% CI)  Specificity：93.7% (95% CI) PPV ：58.8% (95% CI) | ^43^ |
| LN | PHACTR4* | serum | ELISA | AUC:0.99 ,PHACTR4 ICx* were significantly elevated | ^44^ |
| LN | P3H1* | serum | ELISA | AUC: 0.82, P3H1 ICx was found significantly downregulated in LN | ^44^ |
| LN | RGS12* | serum | ELISA | AUC:0.90,RGS12 ICx was found upregulated in LN | ^44^ |
| j-NPSLE | neopterin | CSF* | liquid chromatography | Neopterin levels were significantly elevated in both active and inactive NPSLE patients. | ^45^ |
| j-NPSLE | IFN-α* | CSF | ELISA | It was significantly increased in both active and inactive NPSLE patients. | ^45^ |
| JSLE* | hsa_circ_0008945 | PBMCs | RT-qPCR | AUC=0.790 (95%CI: 0.6733-0.9067, P < 0.001), specificity 83.33%, sensitivity 70% | ^46^ |
| pSLE* | Ang*-1, Ang-2, and Tie2 | Serum  urine | ELISA | The AUC values of Ang-1, Ang-2 and Tie2 in serum and urine were all greater than 0.7. | ^47^ |
| cSLE | IFI44L* promoter methylation | blood | HRM-qPCR | AUC=0.867, specificity 1.000, sensitivity 0.753 . | ^48^ |
| NPSLE* | sNfL* | serum | SiMoA* | AUC =0.646 (95% CI: 0.554-0.738, p=0.003) | ^49^ |
| NPSLE | α-Klotho* | CSF | ELISA | AUC = 0.94 (p < 0.001) | ^50^ |
| NPSLE | L-Selectin* | CSF | 1000-plexed proteins array | sensitivity: 62.16%; specificity: 72.22% | ^51^ |
| NPSLE | Trappin-2 | CSF | 1000-plexed proteins array | sensitivity: 89.19%; specificity: 66.67% | ^51^ |
| NPSLE | TCN2 | CSF | 1000-plexed proteins array | AUC=0.703, P=0.0155 and sensitivity: 54.05%; specificity: 88.89% | ^51^ |
| NPSLE | CST6 | CSF | 1000-plexed proteins array | AUC=0.738, P=0.0043 and sensitivity: 51.35%; specificity: 94.44% | ^51^ |
| NPSLE | MAP-2* | CSF | ELISA | The positive rate of anti-MAP-2 antibody in NPSLE was 33.3% (8/24), the positive rate of CSF showed high specificity for NPSLE. | ^52^ |
| SLE‐PAH* | GDF‐15* | serum | enzyme‐linked immunosorbent assay | AUC=0.84, when GDF-15 ≥733 pg/mL, the specificity and sensitivity of SLE-PAH diagnosis were 58.6% and 91%. | ^53^ |
| IMN* | PLA2R-AB* | serum | ELISA | ROC curves of the PLA2R-AB showed that AUC: 0.907, Sensitivity 94.3%, Specificity 82.1%. | ^54^ |
| CVD* | Lactosylceramides | plasma | HPLC-MS/MS* | Lactosylceramides Correlate Negatively With Plaque Area and C3 | ^55^ |

*PCR: polymerase chain reaction; AUC: area under the ROC curve; CI: chronicity index; DDX60:DExD/H-Box helicase 60; RT-PCR:Reverse-transcription PCR; ELISA: enzyme-linked immunosorbent assay; PPV: positive predictive value; NPV: negative predictive value; HC: healthy controls; NRGs: NETs-related genes, NETs:Neutrophil extracellular traps; WB: whole blood; PBMC: peripheral blood mononuclear cell; qRT-PCR: Real-time reverse transcription PCR; Sema4A: Semaphorin 4A; SLEDAI: systemic lupus erythematosus disease activity index; sTREM-1: soluble Triggering receptors expressed on myeloid cell-1; ROC: Receiver Operating Characteristic; RGC‑32: Response gene to complement‑32; His: histidine; RNA-seq: Ribonucleic acid sequencing; DifCir: differential analysis of eccDNA; cf-eccDNA: Cell-free (cf) extrachromosomal circular DNA (eccDNA); eccDNA: extrachromosomal circular DNA; DNASE1L3: deoxyribonuclease 1-like 3; IFIT3: interferon-induced protein with tetratricopeptide repeats 3; MX1: GTPbinding protein Mx1; TOMM40: mitochondrialimport receptor subunit TOM40 homolog ; STAT1: Signal transducer and activator of transcription 1 ; STAT2: Signal transducer and activator of transcription 2; OAS3: 2’-5’-oligoadenylate synthase 3; S100A8: S100 calcium-binding protein A8 protein; RT-qPCR: reverse transcription quantitative polymerase chain reaction ; tsRNAs: tRNA-derived small noncoding RNA; sTREM-1: soluble triggering receptor expressed on myeloid cells-1; C3: complement C3; C4: complement C4; VSIG4: V-set immunoglobulin domain–containing protein 4; BCDF: B cell differentiating factor; IgM: immunoglobulin M; GDF-15: Growth differentiation factor 15; MX2: MX Dynamin Like GTPase 2; IFI44: interferon induced protein 44; PHACTR4: phosphatase and actin regulator 4; P3H1: prolyl 3-hydroxylase 1; RGS12: regulator of G-protein signaling 12; ICx: immune complexes; j-SLE: Juvenile systemic lupus erythematosus; IFN-ɑ: interferon-alpha; CSF: cerebrospinal fluid; sNfL: serum neurofilament light chain; SiMoA: single molecule array; NPSLE: neuropsychiatric systemic lupus erythematosus; α-Klotho: single-pass transmembrane protein ɑ-Klotho; GDF‐15: Growth‐differentiation factor (GDF)‐15; SLE‐PAH: systemic lupus erythematosus‐associated pulmonary arterial hypertension; IMN: idiopathic membranous nephropathy; PLA2R-AB: phospholipase A2 receptor autoantibodies; CVD: cardiovascular disease; HPLC-MS/MS: high performance liquid chromatography-tandem mass spectrometry;pSLE: Pediatric-onset SLE; Ang: angiopoietins; Tie2: tyrosine kinase receptor; IFI44L: IFN-induced protein 44-like; MINA: Myc-induced nuclear antigen; CSF proteins: TCN2, CST6, L-selectin, Trappin-2; MAP-2: anti-microtubule associated protein 2; SVM: Support vector machine.

**TABLE 2** | Emerging activity markers of SLE.

| **Organ-**  **Specific** | **Marker** | **Specimen** | **method** | **Key Points** | **Reference** |
| --- | --- | --- | --- | --- | --- |
|  | CD137 | PBMCs*  serum | ELISA | Serum sCD137 level is positively correlated with the percentage of CD4+CD137+ cells, which can be used as a biomarker of disease activity. | ^56^ |
|  | PD-1* | PBMC | ELISA | The expressions of PD-1, PD-L1* and sPD-1* were negatively correlated with SLE disease activity and could be used as potential biomarkers for SLE. | ^57^ |
|  | IFN* | serum | RT-PCR | Serum IFN activity was significantly increased in SLE patients. There was a moderate positive correlation with the total score of EULAR/ACR-2019. | ^58^ |
|  | Anti-dsDNA* | PBMCs serum | the SLE-ELISpot assay | When SLE-ELISpot activity critical value (≥11.24 points), the sensitivity was 57.1%, the specificity was 83.9%. | ^59^ |
|  | Platelet LGALS3BP* | platelet | RNA-seq | Platelet release levels of LGALS3BP are highly correlated with circulating LGALS3BP (associated with disease severity) | ^60^ |
|  | PHACTR2*, GOT2*,  SELL*, CMC4*, MAP2K1*, CMPK2*, ECPAS*, SRA1*, STAT2* | PBMC | ELISA | AUC =0.990 (95% CI = 0.968-1) | ^29^ |
|  | Anti-GAPDH* | serum | ELISA | It was positively correlated with SLEDAI-2K, ESR, IgG and IgM. | ^61^ |
|  | AGR* | blood | ordinal logistic regression analysis | AGR (β = −1.319, 95% confidence interval [CI] –2.595, –0.042; P = 0.043) is an independent risk factor for SLE disease activity. | ^62^ |
|  | lncRNA panel (MIR31HG, NKILA) |  | qRT-PCR | It was significantly correlated with albumin/creatinine ratio, glomerular filtration rate and SLEDAI score. | ^63^ |
|  | AGEs* | skin | skin autofluorescence | The AGEs of SLE was 0.721, higher than that of healthy control group. | ^64^ |
|  | T cell expressions of aberrant gene signatures and Co-IRs* | PBMC | ELISA | It is associated with disease activity, nephritis, and response to treatment in patients with SLE. | ^65^ |
|  | PLR* |  | meta-analyses | PLR (SMD = 0.604, 95% CI = 0.299-0.909, p = 0.001) in SLE patients was significantly higher than normal. | ^66^ |
|  | gene EPSTI1 | PBMCs | RT-qPCR | In patients with SLE, EPSTI1 was positively associated with disease activity and T cell-related genes. | ^67^ |
| LN | IGBP1* | serum | ELISA | The specificity and sensitivity of serum IGBP1 level (critical value 547.45 ng/mL) were 96.9% and 93.8%, which can be used as biomarkers for active nephritis. | ^68^ |
| LN | CD56brightCD16- to CD57+CD56dimCD16+ NK cell ratio | blood,  serum | Flow cytometry | The AUC for patients with severe SLE was 0.722, with an optimal cut-off of 0.075, and the ratio for patients with LN was 0.773, with an optimal cut-off of 0.108. | ^69^ |
| LN | usCD163* | Blood,  urine | ELISA | usCD163 levels were significantly elevated in patients with active LN and correlated with UPCR, disease activity, and anti-DSDNA Ab levels. | ^70^ |
| LN | uNGAL*, uKIM*, uNGAL/Creat ratio, and uKIM/Creat ratio | Urine | ELISA | When uNGAL level is above 59, the specificity and sensitivity of diagnosis of active nephritis are 100%, 95%, AUC=0.996. In addition, at levels above 1.6, its specificity is 80%, sensitivity is 95%, and AUC=0.919. | ^71^ |
| LN | MX2 | whole blood and peripheral blood | qRT-PCR | MX2 is highly expressed in SLE and positively correlated with SLE disease severity and SLEDAI. | ^41^ |
| LN | sALCAM* | urine | ELISA | correlation coefficients ranging from 0.35 to 0.41 | ^72^ |
| LN | VCAM-1* | urine | ELISA | AUC ：0.81, P =0.009 ，sensitivity and specificity vales ranging from 78-92% | ^73^ |
| LN | ALCAM* | urine | ELISA | AUC 0.75，P = 0.0001，sensitivity and specificity vales ranging from 78-92% | ^73^ |
| LN | PF4* | urine | ELISA | AUC 0.778，P = 0.001 | ^73^ |
| LN | Ungal*, uKIM-1* | serum | ELISA | When uNGAL level is greater than 59, the sensitivity and specificity of LN detection are 95% and 100%. When the uNGAL/ creatinine ratio is greater than 92, the sensitivity and specificity are 100% and 97%. | ^74^ |
| LN | Nrf2* | blood | Kruskal-Wallis test | Serum Nrf2 protein levels in LN patients were positively correlated with kidney injury and SLEDAI. | ^75^ |
| LN | NLR* | blood | Kruskal-Wallis test | The NLR specificity was 75%, the sensitivity was 71.9%, the AUC=0.747. | ^76^ |
| LN | PLR* | blood | Kruskal-Wallis test | The PLR sensitivity was 87.5%, the specificity was 50%, and the AUC was 0.658. | ^76^ |
| LN | SIRI* | blood | Kruskal-Wallis test | SIRI has an AUC of 0.627, an optimal cut-off of 1.225 (p=0.081), a specificity of 62.5%, and a sensitivity of 65.6%. | ^76^ |
| LN | SII* | blood | Kruskal-Wallis test | The AUC of SII was 0.708, the specificity was 0.750, and the sensitivity was 0.719. | ^76^ |
| ACLE* | TNF-α* |  | logistic regression analysis | AUC=0.716 | ^77^ |
| cSLE | hemopexin | urine | ELISA | AUC: 0.81 , P <0.0001 | ^73^ |
| NPSLE | IL-6* | CSF | retrospectively analyzed | The IL-6 level of CSF before treatment was 29.1 pg/mL, which was significantly decreased compared with 3.8 pg/mL after treatment  (p = 0.008). | ^78^ |
| NPSLE | HMGB1 | serum | ELISA | a significant correlation between HMGB1 levels and SLEDAI-2k (r=0.6527, P=0.000), AUC（95%CI）:0.843（0.783-0.903）OR: 1.7 | ^79^ |

*PBMCs: Peripheral Blood Mononuclear Cells; PD-1: programmed death-1; PD-L1: programmed death ligand-1; sPD-1: the soluble form of PD-1; IFN: interferon; anti-dsDNA: anti-double-stranded DNA; LGALS3BP: soluble 3 binding protein; PHACTR2: phosphatase and actin regulator 2; GOT2: glutamate oxaloacetate transaminase 2 ; SELL: L-selectin ; CMC4: Cx9C motif-containing protein 4 ; MAP2K1: dual specificity mitogen-activated protein kinase kinase 1; CMPK2: cytidine/uridine monophosphate kinase 2 ; ECPAS: Ecm29 proteasome adaptor and scaffold ; SRA1: steroid receptor RNA activator 1; STAT2: transcription 2; anti-GAPDH: glyceraldehyde 3-phosphate dehydrogenase autoantibodies ; IGBP1: Immunoglobulin-binding protein 1; usCD163: Urine-soluble CD163; uKIM: Urinary Kidney Injury Molecule; uNGAL: Urinary neutrophil gelatinase‑associated lipocalin; uKIM‑1: Urinary kidney injury molecule‑1; sALCAM: soluble activated leukocyte cell adhesion molecule; VCAM-1: vascular CAM-1,CAM: cell adhesion molecule; ALCAM: activated leukocyte CAM; PF4: platelet factor-4; IL-6: Interleukin-6; uNGAL: Urinary neutrophil gelatinase‑associated lipocalin; uKIM-1: kidney injury molecule-1; Nrf2: nuclear factor erythroid 2-related factor 2; NLR: neutrophil/lymphocyte ratio; PLR: platelet/lymphocyte ratio; SIRI: systemic inflammatory response index; SII: systemic immuneinflammatory index; TNF-α: tumor necrosis factor-alpha; ACLE: Acute cutaneous lupus erythematosus; AGR: albumin-to-globulin ratio; AGEs: Advanced glycation end-products; Co-IRs: Co-inhibitory receptors; PLR: the platelet-to-lymphocyte ratio.

**TABLE 3** | Other potential SLE markers.

| **Organ-**  **Specific** | **Marker** | **Specimen** | **method** | **Key Points** | **Reference** |
| --- | --- | --- | --- | --- | --- |
|  | GAPDH | serum | ELISA | AU = 83.07 ( 43.66-115.8 ) in NPSLE group and AU = 68.46 ( 46.48-93.81 ) in non-NPSLE group, P = 0.0588. | ^61^ |
|  | miR-101-3p* | PBMC | ELISA | The level of miR-101-3p in PBMC cells in SLE was significantly decreased. | ^80^ |
|  | m6A* | PBMC | RT-qPCR | The alteration of m6A modification in SLE can affect the inflammatory process and participate in the pathogenesis of SLE. | ^81^ |
|  | C3 level | serum | ELISA | When the cut-off value was ≥0.780 g/L, the specificity was 75.0% and the sensitivity was 70.6% , AUC = 0.756 ( p = 0.003). | ^82^ |
| LN | SUA | serum | retrospective observational study | SUA in LN is associated with renal severity. | ^83^ |
| LN | Serum salusin-β | serum | ELISA | salusin-β in SLE group was 474.2±117.1 pg/ml, which was significantly higher than that in healthy group (157.7±88.7 pg/ml). (P = 0.001) | ^84^ |
| LN | NET* remnants (Elastase-DNA and HMGB1-DNA complexes) | serum | ELISA | NET remnants is significantly increased in LN and can be used as a marker to identify poor prognosis. | ^85^ |
| LN | OPN* | serum | ELISA | OPN in LN was higher than that in normal people or SLE patients with other organ involvement (p<0.0001 and 0.0032, respectively), independent of renal activity, and correlated with low complement levels in LN. | ^86^ |
| NPSLE | AECA* | serum | ELISA | AECA was found in 11 ( 64.7 % ) of 17 NPSEL patients and 10 ( 29.4 % ) of 34 non-NP SLE patients | ^87^ |
| NPSLE | Autoantibodies to TPI* | serum | ELISA | Ten of the 31 NPSLE patients had anti-TPI positivity (32.3%),Sensitivity 32.3%, specificity 95% for NPSLE | ^88^ |
| LE* | Anti-gAChRα3 Abs* | serum | luciferase immunoprecipitation system assay. | The odds ratio of anti-Gachrα3 antibody was 6.2 (95%CI: 1.9-20.3, p = 0.002), and it could be used as a predictor of LE. | ^89^ |
| Fatigue with SLE | NR2( NMDAR* subunit) | serum | ELISA | Anti-NR2 antibodies were detected in 46.9 % of SLE patients with fatigue ( titer > 2 ng / mL ). | ^90^ |
| CVD | NT-proBNP* | serum | multivariable logistic regression model | The AUC of NT-proBNP =0.78 (95%CI: 0.69-0.87), with a threshold of 133 pg/ml, was strongly correlated with CVD. | ^91^ |

(Markers related to SLE severity, occurrence, development, occurrence or recurrence, and treatment)

*miR-101-3p: microRNA-101-3p; SUA: serum uric acid; OPN: Osteopontin; LE: Lupus enteritis; Anti-gAChRα3 Abs: anti-ganglionic nicotinic acetylcholine receptor α3 subunit (gAChRɑ3) antibodies (Abs); NMDAR: antiN-methyl-D-aspartate receptor; NT-proBNP: N-terminal pro-brain natriuretic peptide; NET: neutrophil extracellular trap; m6A: N6-methyladenosine; AECA: anti-endothelial-cell antibodies; TPI: triosephosphate isomerase .
