# Supplementary material for: Biomarkers for systemic lupus erythematosus: A scoping review
Source: Immun Inflamm Dis. 2024 Oct 4;12(10):e70022. doi: 10.1002/iid3.70022 (PMC11450456; doi:10.1002/iid3.70022)
Supplement: Supplementary file 2 — Supporting information. [file IID3-12-e70022-s001.docx]

Full definitions for all terms

SLE: systemic lupus erythematosus;

ESKD: end-stage kidney disease;

LN: Lupus Nephritis;

SLEDAI: Systemic Lupus Erythematosus Disease Activity Index ;

MAC: membrane attack complex;

AUC: The area under the ROC curve ;

TNF-RI : Tumor necrosis factor receptor I;

TNF-α: tumor necrosis factor-α;

ROC: The Receiver Operating Characteristic;

IL-23: Interleukin-23 ;

IL-12: Interleukin-12 ;

b2MG: Beta-2 microglobulin ;

MHC-I: major histocompatibility complex class I ;

PPV: positive predictive value;

NPV: negative predictive value ;

JSLE: juvenile SLE;

Cys C: Cystatin C;

MPO-DNA: myeloperoxidase–deoxyribonucleic acid;

VSIG4: V-set immunoglobulin domain–containing protein 4;

AI: activity index;

Gal-3BP: Galectin-3-binding protein;

u-Gal-3BP: Urinary galectin-3 binding protein;

ANR-SLE: active non-renal SLE;

SLEDAI: Systemic Lupus Erythematosus Disease Activity Index;

cSLE: Childhood-onset systemic lupus erythematosus;

tsRNAs: The tRNA-derived small RNAs;

tRFs: tRNA-derived fragments;

tiRNAs: tRNA halves ;

mCRP: Modified C-reactive protein;

u-KIM-1: urinary (u)-kidney injury molecule-1;

sTREM-1: soluble triggering receptor expressed on myeloid cells-1;

ARL: active renal lupus;

lncRNA: long non-coding RNA;

CASC2: cancer susceptibility candidate 2;

MALAT1: metastasis-associated lung carcinoma transcript 1;

TUG1: taurine-upregulated gene 1;

eGFR: estimated glomerular filtration rate;

MCN: minimal change nephropathy;

PTC-C4d: Peritubular capillary C4d;

IFI16: Interferon-inducible protein 16;

WGCNA: Weighted gene coexpression network analysis;

IFN: interferon;

NPSLE: Neuropsychiatric In SLE;

IL: interleukin;

CSF: cerebrospinal fluid;

β_2_GPI: anti-β_2_-glycoprotein I;

Ig: immunoglobulin;

OR: odds ratio;

CI: confidence interval;

HMGB1: high‐mobility group box protein 1;

TNF-α: tumor necrosis factor- alpha;

IFN-γ: interferon-gamma;

CCL: C-C motif ligand 2;

MCP-1: monocyte chemoattractant protein-1;

CXCL: C-X-C motif ligand 10;

IP-10: inducible protein-10;

CLE: cutaneous lupus erythematosus;

ANA: antinuclear antibody;

CVD: Cardiovascular disease;

pDCs: plasmacytoid dendritic cells;

PCR: polymerase chain reaction;

CI: chronicity index;

DDX60:DExD/H-Box helicase 60;

RT-PCR:Reverse-transcription PCR;

ELISA: enzyme-linked immunosorbent assay;

HC: healthy controls;

NRGs: NETs-related genes,

NETs:Neutrophil extracellular traps;

WB: whole blood;

PBMC: peripheral blood mononuclear cell;

qRT-PCR: Real-time reverse transcription PCR;

Sema4A: Semaphorin 4A;

SLEDAI: systemic lupus erythematosus disease activity index;

RA: rheumatoid arthritis;

sTREM-1: soluble Triggering receptors expressed on myeloid cell-1;

RGC‑32: Response gene to complement‑32;

His: histidine;

RNA-seq: Ribonucleic acid sequencing;

DifCir: differential analysis of eccDNA;

cf-eccDNA: Cell-free (cf) extrachromosomal circular DNA (eccDNA);

eccDNA: extrachromosomal circular DNA;

DNASE1L3: deoxyribonuclease 1-like 3;

IFIT3: interferon-induced protein with tetratricopeptide repeats 3;

MX1: GTPbinding protein Mx1;

TOMM40: mitochondrialimport receptor subunit TOM40 homolog ;

STAT1: Signal transducer and activator of transcription 1 ;

STAT2: Signal transducer and activator of transcription 2;

OAS3: 2’-5’-oligoadenylate synthase 3;

S100A8: S100 calcium-binding protein A8 protein;

RT-qPCR: reverse transcription quantitative polymerase chain reaction ;

tsRNAs: tRNA-derived small noncoding RNA;

sTREM-1: soluble triggering receptor expressed on myeloid cells-1;

C3: complement 3;

C4: complement 4;

VSIG4: V-set immunoglobulin domain–containing protein 4;

BCDF: B cell differentiating factor;

IgM: immunoglobulin M;

GDF-15: Growth differentiation factor 15;

MX2: MX Dynamin Like GTPase 2;

IFI44: interferon induced protein 44;

PHACTR4: phosphatase and actin regulator 4;

P3H1: prolyl 3-hydroxylase 1;

RGS12: regulator of G-protein signaling 12;

ICx: immune complexes;

j-SLE: Juvenile systemic lupus erythematosus;

IFN-ɑ: interferon-alpha;

CSF: cerebrospinal fluid;

sNfL: serum neurofilament light chain;

SiMoA: single molecule array;

NPSLE: neuropsychiatric systemic lupus erythematosus;

α-Klotho: single-pass transmembrane protein ɑ-Klotho;

GDF‐15: Growth‐differentiation factor (GDF)‐15;

SLE‐PAH: systemic lupus erythematosus‐associated pulmonary arterial hypertension;

IMN: idiopathic membranous nephropathy;

PLA2R-AB: phospholipase A2 receptor autoantibodies;

CVD: cardiovascular disease;

HPLC-MS/MS: high performance liquid chromatography-tandem mass spectrometry.

PBMCs: Peripheral Blood Mononuclear Cells;

PD-1: programmed death-1;

PD-L1: programmed death ligand-1;

IFN: interferon; anti-dsDNA: anti-double-stranded DNA;

LGALS3BP: soluble 3 binding protein;

PHACTR2: phosphatase and actin regulator 2;

GOT2: glutamate oxaloacetate transaminase 2 ;

SELL: L-selectin ;

CMC4: Cx9C motif-containing protein 4 ;

MAP2K1: dual specificity mitogen-activated protein kinase kinase 1;

CMPK2: cytidine/uridine monophosphate kinase 2 ;

ECPAS: Ecm29 proteasome adaptor and scaffold ;

SRA1: steroid receptor RNA activator 1;

STAT2: transcription 2;

anti-GAPDH: glyceraldehyde 3-phosphate dehydrogenase autoantibodies ;

IGBP1: Immunoglobulin-binding protein 1;

usCD163: Urine-soluble CD163;

uKIM: Urinary Kidney Injury Molecule;

uNGAL: Urinary neutrophil gelatinase‑associated lipocalin;

uKIM‑1: Urinary kidney injury molecule‑1;

sALCAM: soluble activated leukocyte cell adhesion molecule;

VCAM-1: vascular CAM-1, CAM: cell adhesion molecule;

ALCAM: activated leukocyte CAM;

PF4: platelet factor-4;

IL-6: Interleukin-6;

miR-101-3p: microRNA-101-3p;

SUA: serum uric acid;

OPN: Osteopontin;

LE: Lupus enteritis;

Anti-gAChRα3 Abs: anti-ganglionic nicotinic acetylcholine receptor α3 subunit (gAChRɑ3) antibodies (Abs);

NMDAR: antiN-methyl-D-aspartate receptor;

pSLE: Pediatric-onset SLE;

Ang: angiopoietins;

Tie2: tyrosine kinase receptor;

IFI44L: IFN-induced protein 44-like;

MINA: Myc-induced nuclear antigen;

CSF proteins: TCN2, CST6, L-selectin, Trappin-2;

MAP-2: anti-microtubule associated protein 2;

uNGAL: Urinary neutrophil gelatinase‑associated lipocalin;

uKIM-1: kidney injury molecule-1;

Nrf2: nuclear factor erythroid 2-related factor 2;

NLR: neutrophil/lymphocyte ratio;

PLR: platelet/lymphocyte ratio;

SIRI: systemic inflammatory response index;

SII: systemic immuneinflammatory index;

TNF-α: tumor necrosis factor-alpha;

ACLE: Acute cutaneous lupus erythematosus;

AGR: albumin-to-globulin ratio;

AGEs: Advanced glycation end-products;

Co-IRs: Co-inhibitory receptors;

PLR: the platelet-to-lymphocyte ratio;

NT-proBNP: N-terminal pro-brain natriuretic peptide;

NET: neutrophil extracellular trap;

m6A: N6-methyladenosine;

AECA: anti-endothelial-cell antibodies;

TPI: triosephosphate isomerase ;

BCR: The B cell receptor;

NETs: neutrophil extracellular traps;

TLR7: Toll-like receptor 7 .
